# Development of an LC–MS/MS Assay and Toxicokinetic Characterization of Hexamethylenetetramine in Rats

**DOI:** 10.3390/toxics11040337

**Published:** 2023-03-31

**Authors:** Woojin Kim, Eunbin Kim, Jaewoong Lee, Chang Ho Song, Woohyung Jung, Soyoung Shin, Kyu-Bong Kim, Beom Soo Shin, Tae Hwan Kim

**Affiliations:** 1 College of Pharmacy, Daegu Catholic University, Gyeongsan 38430, Republic of Korea; 2 School of Pharmacy, Sungkyunkwan University, Suwon 16419, Republic of Korea; 3 College of Pharmacy, Wonkwang University, Iksan 54538, Republic of Korea; 4 College of Pharmacy, Dankook University, Cheonan 31116, Republic of Korea

**Keywords:** hexamethylenetetramine, methenamine, toxicokinetics, LC–MS/MS, preservative

## Abstract

Hexamethylenetetramine, an aldehyde-releasing agent, is used as a preservative in various food, cosmetics, and medical treatments, such as a treatment for urinary tract infections. It has been reported to be allergenic on contact with the skin, with the additional possibility of causing toxicity once absorbed systemically. Despite its potential toxicity, there are no reports on the in vivo bioavailability of hexamethylenetetramine following oral or dermal administration. In this study, we developed a new simple and sensitive LC–MS/MS method for the determination of hexamethylenetetramine in plasma and applied this method to characterize the toxicokinetics. The developed assay had a sufficient specificity and sensitivity for toxicokinetic characterization, and its accuracy and precision were verified. Following iv injection, the plasma concentration of hexamethylenetetramine showed mono exponential decay, with an elimination half-life of about 1.3 h. Following oral administration, the T_max_ reached an average of 0.47 h and bioavailability was estimated as 89.93%. After percutaneous administration, it reached C_max_ on average at 2.9–3.6 h. Although the absorption rate was relatively slow, its average bioavailability was calculated as 77.19–78.91%. Overall, most of the orally and percutaneously administered hexamethylenetetramine was absorbed into systemic circulation. The derived results in this study are expected to be utilized as the scientific evidence for further toxicokinetic study and risk assessment.

## 1. Introduction

Hexamethylenetetramine, also known as methenamine, is one a preservative commonly used in various food, medical, and cosmetic applications. It is known that the antimicrobial activity of hexamethylenetetramine is attributed to the production of formaldehyde that is released by the hydrolysis of hexamethylenetetramine under acidic or thermal conditions. Thus, hexamethylenetetramine is used as a preservative in processed meat, dairy products, canned foods, and beverages such as beer and wine. It is also added to cosmetic products such as shampoos, lotions, and creams. Moreover, hexamethylenetetramine has been also used as an anti-bacterial agent to prevent and control recurrent or chronic urinary tract infections [1,2,3,4,5].

For urinary tract infections in adults, 1 g of hexamethylenetetramine hippurate salt or mandelate salt is administered twice or four times daily, respectively [6,7]. The toxicokinetics of hexamethylenetetramine were characterized in humans, obtained after the oral administrations of granule and tablet formulations containing 1 g of hexamethylenetetramine hippurate. Following oral administration, maximum blood concentration was observed about 1–2 h after dosing and elimination half-life (t_1/2_) was calculated as 4 h. About 80% of the dosed hexamethylenetetramine was excreted via urine [8,9]. Therefore, high bioavailability following oral administration of hexamethylenetetramine is expected, however, the exact absolute bioavailability of the compound has not been reported to date.

Following oral administration of hexamethylenetetramine as a medication, some adverse effects related to gastrointestinal disturbance including abdominal pain, nausea, vomiting, and diarrhea have been reported. In humans, about 10–30% of the orally administered hexamethylenetetramine is reported to be converted to formaldehyde and ammonia under the acidic pH of the stomach. Due to the presence of ammonia in the gastrointestinal tract, those gastrointestinal adverse effects would occur. As described, a significant portion of orally administered hexamethylenetetramine is absorbed, and related systemic adverse events including painful and frequent micturition and hematuria have been reported. Since the urine is relatively acidic, formaldehyde, which is responsible for the anti-bacterial activity, is released from hexamethylenetetramine. However, these side effects also occur from the released formaldehyde [10,11].

As such, most of the toxicity and toxicokinetics of hexamethylenetetramine were characterized following oral administrations. Recently, it has been reported that hexamethylenetetramine can cause allergic contact dermatitis following topical application [1,12,13,14]. According to some case reports, allergic contact dermatitis is considered to be caused by hexamethylenetetramine itself or released formaldehyde [15,16]. Since orally administered hexamethylenetetramine is well-absorbed, a significant amount may be transferred to systemic circulation once the compound penetrates the stratum corneum. In this case, those systemic activities, as well as allergic dermatitis, can be caused by applying consumer products containing hexamethylenetetramine.

In several countries, hexamethylenetetramine has been managed as an ingredient in consumer products requiring restrictions on its use. Its maximum allowable concentration is regulated at 0.15% in EU and Korea [17,18]. In some countries, such as Taiwan and China, its use as a cosmetic ingredient is inhibited [19,20].

Concerning the unintended dermal permeation potential of hexamethylenetetramine, there is no report about its systemic absorption and bioavailability following percutaneous administration in humans or animals at present. Moreover, reliable analytical assays for the determination of hexamethylenetetramine in a biological matrix in order to utilize toxicokinetic studies have not been reported. Although some analytical assays for the determination of hexamethylenetetramine have been reported, their sensitivities are not sufficient or sample preparation procedures are too complicated [10,11,21].

Several analytical methods for the quantification of hexamethylenetetramine in urine by converting it to formaldehyde have been reported. These assays indirectly determined the hexamethylenetetramine in human urine by quantification of formaldehyde concentration converted by sample preparation [22,23,24,25]. There were several limitations such as complex extraction procedures, insufficient sensitivity, and poor selectivity with pre-released formaldehyde.

A method for directly quantifying hexamethylenetetramine in human serum and urine using gas chromatography (GC) coupled with nitrogen selective detector has also been reported. Hexamethylenetetramine in serum was extracted using acetone. The LLOQ of hexamethylenetetramine in serum and urine was 0.6 and 50 μg/mL, respectively [21]. Later, this assay was slightly modified and utilized for human toxicokinetics following oral administration of 1 g of hexamethylenetetramine hippurate salt [9]. Nevertheless, the sensitivity is not considered sufficient enough for the determination of hexamethylenetetramine in biological matrices obtained after the percutaneous administration of cosmetics containing hexamethylenetetramine.

Risk assessment is a process to determine whether exposure to an amount of specific products causes hazards, or is considered safe. For this process, the margin of safety (MoS) has to be calculated by comparing the systemic exposure dosage (SED) and no observed adverse effect level (NOAEL) [26]. The MoS can be calculated as (1).

MoS = NOAEL/SED(1)

In general, the NOAEL is estimated by repeated dose toxicity studies. To calculate the SED, toxicokinetic characteristics including bioavailability should be investigated following proper exposure route administration. According to a risk assessment report on hexamethylenetetramine, its risk characterization was carried out [11]. To calculate MoS, a NOAEL of 57 and 27 mg/kg/day was adopted for repeated dose toxicity and developmental toxicity, respectively. For the evaluation of SED, the amount of cosmetics containing 0.15% HMTA was set at 0.445 mg/kg in normal consumers. Bioavailabilities of hexamethylenetetramine for oral, dermal, and respiratory exposure were assumed as 100, 50, and 100%, respectively. For oral exposure, bioavailability was assumed based on previously reported data. On the other hand, those for dermal and respiratory exposure were assumed by the physicochemical properties of hexamethylenetetramine. To date, since there is no report for toxicokinetic information such as bioavailability after percutaneous application of hexamethylenetetramine, it is necessary to characterize its toxicokinetics for the estimation of a reliable SED.

Therefore, in the present study, we aimed to develop a simple and sensitive liquid chromatography with tandem mass spectrometry (LC–MS/MS) assay for the quantification of hexamethylenetetramine, and investigate the toxicokinetic properties including absolute bioavailability following oral and percutaneous administrations in rats.

## 2. Materials and Methods

### 2.1. Materials

Hexamethylenetetramine (C_6_H_12_N_4_) and hexamethylenetetramine-_13_C_6_, _15_N_4_ (^13^C_6_H_12_^15^N_4_) were purchased from Sigma-Aldrich Co. (St. Louis, MO, USA). Methanol, water, and acetonitrile (HPLC grade) were purchased from J.T. Baker, inc. (Phillipsburg, NJ, USA. Ammonium formate was purchased from Sigma-Aldrich Co. (St. Louis, MO, USA).

### 2.2. LC–MS/MS

#### 2.2.1. Instruments

Agilent 6430 coupled with an Agilent 1200 HPLC system was used for sample analysis. A total of 5 μL of sample was injected into the instrument with the mobile phase of 5 mM ammonium formate (A):Acetonitrile (B) = 20:80 (*v*/*v*) as flow of 0.5 mL/min. To separate various components in the sample, Luna 3μ HILIC 200 Å (150 × 2 mm, Phenomenex, Torrance, CA, USA) was used. Then, the column oven temperature set to 30 °C and run time was 5 min. To detect analytes, a mass spectrometer was used. Polarity of analytes was charged to positive through electron spray ionization (ESI). Transitions of precursor to product ion were 141.0 → 112.0 for hexamethylenetetramine and 151.1 → 120.0 for internal standard with a dwell time of 80 ms. As source parameters, gas temperature, gas flow, and nebulizer were 350 °C, 10 L/min, and 35 psi, respectively. Respective fragmentor voltage and collision energy were set to 66 and 11 V, respectively, for hexamethylenetetramine, and 72 and 11 V, respectively, for internal standard.

#### 2.2.2. Stock and Working Solution

Stock solution of hexamethylenetetramine (1 mg/mL) was prepared by dissolving 10.00 mg in 10 mL of methanol. Working solutions (5, 10, 50, 100, 500, 1000 ng/mL) were prepared by serial dilution of stock solution with acetonitrile. Internal standard stock solution was prepared by dissolving 10.00 mg of hexamethylenetetramine-_13_C_6_, _15_N_4_ in 10 mL of methanol, and the solution was diluted up to 500 ng/mL with acetonitrile. All stock and working solutions were stored at −20 °C.

#### 2.2.3. Preparation of Calibration Standard and Quality Control Samples

By spiking 50 μL of each working standard solution into 50 μL blank rat plasma, calibration standard samples of hexamethylenetetramine were prepared at concentrations of 5, 10, 50, 100, 500, and 1000 ng/mL. Quality control (QC) samples of hexamethylenetetramine were prepared by spiking working standard solutions into blank rat plasma to provide lower limit of quantification (LLOQ, 5 ng/mL), low (15 ng/mL), medium (80 ng/mL), and high (800 ng/mL) QC concentrations. All the calibration standard and QC samples were stored at −20 °C until analysis.

#### 2.2.4. Sample Preparation

For sample preparation, protein precipitation with acetonitrile was utilized. A total of 50 μL of internal standard solution (500 ng/mL) and 2000 μL (150 μL for calibration standards and QC samples) of acetonitrile were added to 50 μL of rat plasma samples. This mixture was vortex-mixed for 1 min and went through centrifugation for 10 min at 4000× *g*. After centrifugation, 150 μL of supernatant of the mixture was transferred to a polypropylene vial and a portion (5 μL) was injected into LC–MS/MS.

#### 2.2.5. Accuracy and Precision

The calibration curves were constructed by the weighted regression method (1/x) of peak area ratios of hexamethylenetetramine to internal standard. The linearity of calibration curves was evaluated with five different calibration curves and repeatability of the method was evaluated through %CV of the slope of calibration curves. Accuracy and precision of the developed assay were evaluated by analyzing 4 replicates each of LLOQ, low, medium, and high QC samples on the same day (intra-day) and 4 consecutive days (inter-day). Accuracy was expressed as a percentage of mean concentration, which was determined by the calibration curve, divided by nominal concentration. Also, precision was expressed as a coefficient of variance at each quality control (QC) concentrations. 

### 2.3. Toxicokinetics of Hexamethylenetetramine

#### 2.3.1. Animals

All animal care and experiments for toxicokinetic characterization were performed according to the Guidelines for the Care and Use of Animal approved by the Ethics Committee for Treatment of Laboratory Animal at Daegu Catholic University (CUD-2018-034). Male Sprague–Dawley rats (7–8 weeks old, body weight 225–250 g) (Hyochang Science, Daegu, Republic of Korea) were kept in plastic cages with free access to a standard diet (Daejong, Seoul, Republic of Korea). Rats were maintained in a facility set to specific conditions, which were at a temperature of 23 ± 2 °C and relative humidity of 50 ± 10% with 12 h light, 12 h dark cycle. The animals were acclimatized for at least 1 week prior to the experiment. 

For serial blood sampling, rats were anesthetized by intra-peritoneal injection (50 mg/kg) of Zoletil 50 (Virbac Laboratories, Carros, France) and polyethylene (PE) tubing (0.58 mm ID and 0.96 mm OD, Natume Co., Tokyo, Japan) was intubated into the jugular vein. The rats were allowed to recover from surgery for at least 36 h. During the study period, one rat was housed per cage. 

#### 2.3.2. IV Injection Study

Hexamethylenetetramine was dissolved in saline for iv injection at doses of 0.2 and 0.5 mg/kg. After overnight fasting, dosing solution was injected via the penile vein. The injected dosing volume was constantly 1 mL/kg regardless of the dose. Blood samples (0.2 mL each) were collected via jugular vein at 0, 2, 5, 10, 15, and 30 min, and 1, 2, 3, 4, 6, 8, 10, 12, and 24 h after injection. Plasma samples were harvested by centrifugation of obtained blood for 10 min at 4000× *g*, and stored at −20 °C until analysis.

#### 2.3.3. Oral Administration Study

Hexamethylenetetramine was dissolved in distilled water prior to oral administration at a dose of 0.5 mg/kg. After overnight fasting, dosing solution was administered using oral zonde. Blood samples (0.2 mL each) were collected via jugular vein at 0, 5, 10, 15, 30, and 45 min, and 1, 2, 3, 4, 6, 8, 10, 12, and 24 h after dosing. Plasma samples were harvested by centrifugation of obtained blood for 10 min at 4000× *g*, and stored at −20 °C until analysis.

#### 2.3.4. Percutaneous Absorption Study

The study was performed according to “OECD Guidelines for the In Vivo Skin Absorption Test” [27]. For the percutaneous absorption study, gel formulation consisting of hexamethylenetetramine (0.15 or 0.6%), carbomer 940 (1.1%), and distilled water (98.75 or 98.3%) was used. About 24 h prior to dermal administration, rats were slightly anesthetized by isoflurane (Hana Pharm Co., Ltd., Seoul, Republic of Korea) and the dorsal site was shaved using electric clippers. The shaved skin surface was carefully cleaned with acetone. After overnight fasting, 5 mg/cm^2^ of hexamethylenetetramine gel was evenly applied to 4.5 × 4.5 cm^2^ of the shaved area. The dose of hexamethylenetetramine was calculated as an average 0.58 and 2.29 mg/kg. The application site was covered by nylon gauze to prevent loss of hexamethylenetetramine from the administration site due to volatilization or grooming. Blood samples (0.2 mL each) were collected via jugular vein at 0, 5, 15, and 30 min, and 1, 1.5, 2, 4, 6, 8, 10, 12, and 24 h after dosing. Plasma samples were harvested by centrifugation of obtained blood for 10 min at 4000× *g*, and stored at −20 °C until analysis.

#### 2.3.5. Data Analysis

Toxicokinetic parameters were calculated by non-compartmental analysis using WinNonlin (Pharsight, Cary, NC, USA) non-linear least squares regression software. The parameters included initial plasma concentration (C_0_), terminal half-life (t_1/2_), systemic clearance (CL), volume of distribution for steady state (Vd_ss_), and area under the plasma concentration–time curve from zero to last observation (AUC_last_), and infinity (AUC_infinity_). The maximum concentration (C_max_) and time to reach that (T_max_) were directly determined by reading observed data. The absolute bioavailability of hexamethylenetetramine was calculated as F (%) (2).

F = AUC_extravascular_ × Dose_i.v._/AUC_i.v._ × Dose_extravascular_ (2)

## 3. Results

### 3.1. Mass Spectrometry and Chromatography

**Figure 1:**
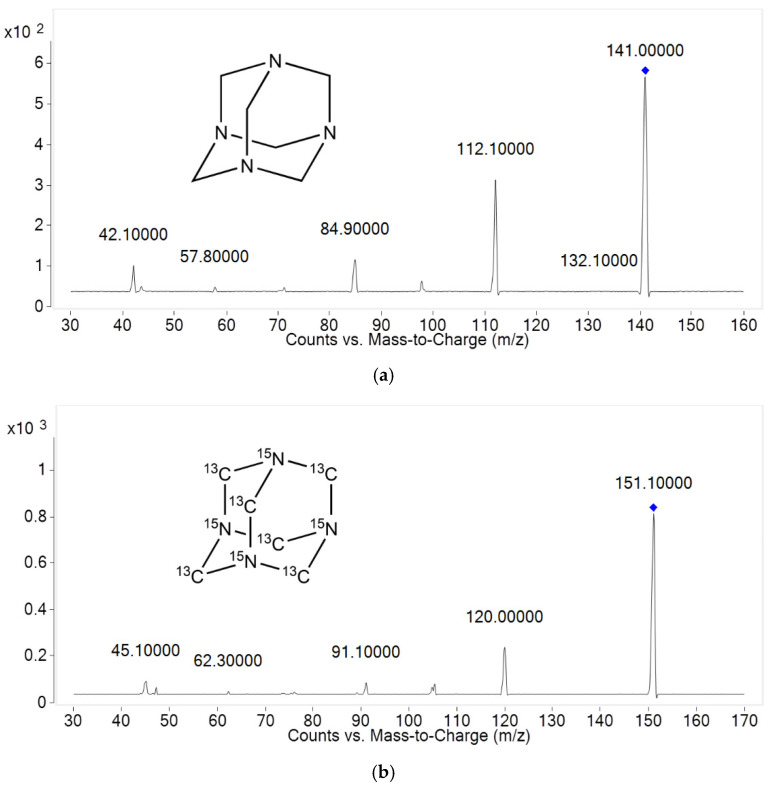
Product ion mass spectra of protonated (**a**) hexamethylenetetramine (141.0 → 112.1) and (**b**) I.S. (151.1 → 120.0) in positive ionization mode.

In this study, a specific and sensitive LC–MS/MS assay was developed for analysis of hexamethylenetetramine in rat plasma. Multi-reaction monitoring (MRM) mass transition was determined as 141.0 → 112.1 for hexamethylenetetramine and 151.1 → 120.0 for internal standard based on the intensity of the protonated ion and the product ion. The product ion mass spectra are presented in Figure 1.

**Figure 2:**
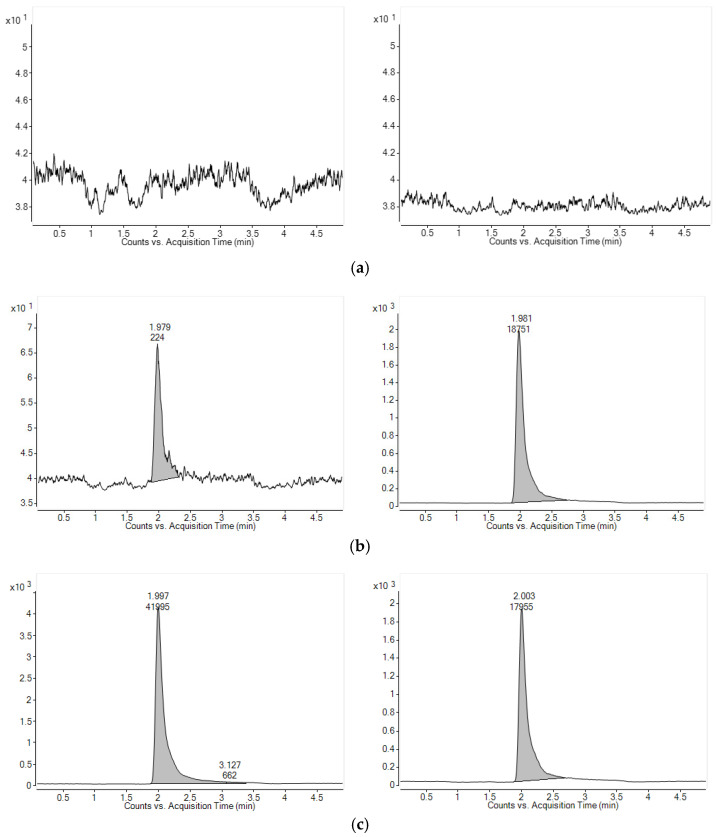
MRM chromatograms of hexamethylenetetramine (**left**) and internal standard (**right**) obtained from (**a**) blank rat plasma, (**b**) LLOQ concentration (5 ng/mL) of hexamethylenetetramine in plasma spiked with hexamethylenetetramine and I.S., and (**c**) ULOQ concentration (1000 ng/mL) of hexamethylenetetramine in plasma spiked with hexamethylenetetramine and I.S.

MRM chromatograms obtained after analyzing double blank (a), LLOQ (b), and ULOQ (c) of hexamethylenetetramine (left) and internal standard (right) are presented in Figure 2. Retention times for both analyte and internal standard were observed as 2.0 min. There were no observed interruptive peaks in the blank plasma at 2.0 min. The S/N ratio of the LLOQ sample was calculated as 75.7, and the shapes of hexamethylenetetramine and IS peaks were acceptable. Overall, the selectivity and sensitivity of the developed analytical method for quantifying hexamethylenetetramine in plasma were confirmed.

### 3.2. Assay Linearity, Accuracy, and Precision

The linearity of the calibration curves was accomplished over the concentration range from 5 to 1000 ng/mL for hexamethylenetetramine, with coefficients of determination (r^2^ > 0.9999). The %CV for the slope of calibration curves used for quantification of hexamethylenetetramine in rat plasma was calculated as 9.47%, indicating the repeatability of the method (Table 1).

Table 2 shows the intra- and inter-day accuracy and precision obtained after analysis of QC samples on four consecutive days with four replicates each day. The intra- and inter-day accuracy for all levels were 92.5–100.0% and 98.4–102.5%, respectively. Also, the intra- and inter-day precisions for all levels were ≤4.3% and ≤4.4%, respectively. All data for the intra- and inter-day accuracy and precision meet the acceptance criteria presented in the FDA and ICH guidelines [28,29].

### 3.3. Toxicokinetics of Hexamethylenetetramine in Rats

**Figure 3:**
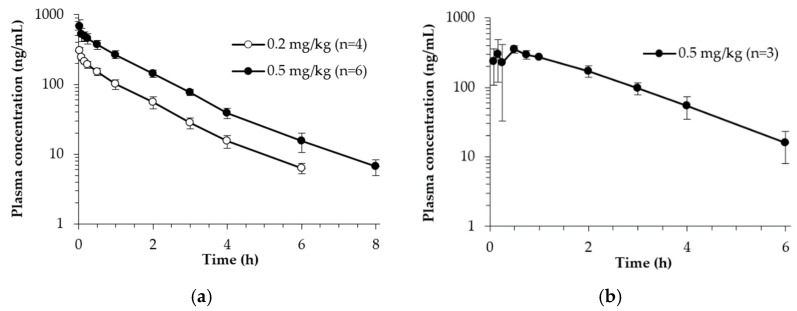

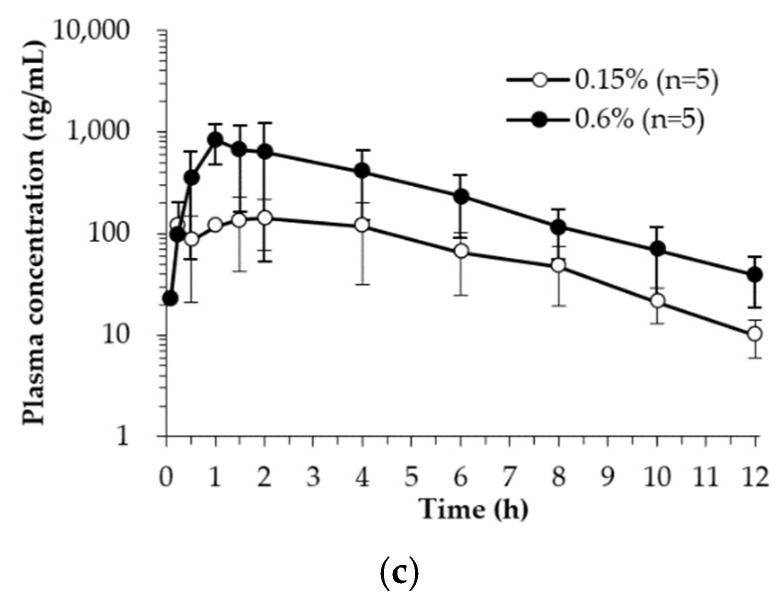
Average plasma concentration–time profiles of hexamethylenetetramine obtained after (**a**) iv injection (0.2 and 0.5 mg/kg), (**b**) oral (0.5 mg/kg), and (**c**) percutaneous administration (0.15 and 0.6%) in rats.

Average plasma concentration–time profiles of hexamethylenetetramine obtained after iv injection, oral administration, and percutaneous administration are shown in Figure 3. After iv injection of 0.2 and 0.4 mg/kg of hexamethylenetetramine, a mono-exponential decline was observed in the plasma concentrations. Following oral administration, hexamethylenetetramine was quantifiable in the first plasma samples (5 min) and reached C_max_ at about 0.5 h. In the percutaneously administered group, hexamethylenetetramine was relatively slowly absorbed. In some animals, 0.5–1 h of lag time was observed. The C_max_ was reached approximately 3 h after dosing.

Average toxicokinetic parameters calculated by non-compartmental analysis are summarized in Table 3 and Table 4. Following iv injection, t_1/2_ was estimated as 1.26–1.36 h and plasma concentration was below that of the LLOQ (BLOQ) at 8 h. The Vd_ss_ and CL were estimated as 0.93–0.95 L/kg and 9.79–9.94 mL/min/kg, respectively.

Orally administered hexamethylenetetramine was detected in plasma at 5–15 min and reaches maximum concentration at 10–45 min. The t_1/2_ was estimated as 1.12 h and absolute bioavailability was calculated as 89.83%.

Following percutaneous administration, hexamethylenetetramine is detected in plasma at 5–15 min and reached C_max_ at 1–6 h. T_1/2_ was estimated as 1.69–2.43 h. The absolute bioavailability was calculated as 78.91 and 77.19% at 0.15 and 0.6% of hexamethylenetetramine gel formulation, respectively. 

## 4. Discussion

The purpose of this study was to develop a new analytical assay for the determination of hexamethylenetetramine in the biological matrix and characterize the toxicokinetics of hexamethylenetetramine in rats. Due to the lack of a reliable LC–MS/MS assay for characterizing the toxicokinetics of hexamethylenetetramine, we developed an analysis method prior to the toxicokinetic study.

In the previous report, complicated and time-consuming extraction was utilized for sample preparation to determine the concentration of hexamethylenetetramine in biological matrices. In the present study, we developed a simple and sensitive analytical method by utilizing protein precipitation. Since hexamethylenetetramine is a highly polar compound (log K_ow_ = −2.18), several chromatographic conditions were tested to separate it from hydrophilic endogenous interferences. The matrix effect was adequately suppressed using reverse phase columns with high aqueous mobile phases, but the assay sensitivity was insufficient. Subsequently, a hydrophilic interaction liquid chromatography column was tested, and acceptable peak shape, sensitivity, selectivity, linearity, and matrix effect were achieved. The developed assay was utilized in the in vivo toxicokinetic study in rats.

Throughout the test period, any signs of toxicity were not observed in rats. Following iv injection, plasma hexamethylenetetramine concentration decreased mono-exponentially. The t_1/2_, Vd_ss_, and CL were not statistically different between dose groups, and C_0_ and AUC increased with the dose increase. That is, the toxicokinetics of hexamethylenetetramine was characterized as linear kinetics in the dose range. In the orally administered group, hexamethylenetetramine is rapidly absorbed, with its concentrations quantifiable in the first plasma samples, and almost 90% of the dosed amount was systemically absorbed. After percutaneous administration, although the absorption rate of hexamethylenetetramine was relatively slow, approximately 80% of the dosed amount of hexamethylenetetramine was systemically absorbed. 

As described above, the main objective of this study was to evaluate the absolute bioavailability of hexamethylenetetramine in rats. Although urine samples were not collected for mass balance estimation, accurate bioavailability was estimated through serial blood sampling. Based on previously published data, it is considered that most of the chemical absorbed into the body is excreted via urine [8,9].

Overall, these results indicate that most of the amount of hexamethylenetetramine is absorbed into the systemic circulation regardless of the route of administration. The bioavailability following extravascular administration is mainly determined by membrane permeability and enzymatic or non-enzymatic degradation during the absorption process. Except for facilitated diffusion, in which substances are transported through specific transporters, passive diffusion is classified into simple diffusion through the transcellular pathway and restricted diffusion through the paracellular pathway. It is generally reported that membrane permeability shows a high correlation with the lipophilicity of a substance [30], which is only applied to simple diffusion in which a substance is dissolved and diffused in a lipid-soluble membrane. Low-molecular-weight and water-soluble chemicals, such as hexamethylenetetramine, are likely to diffuse via the paracellular route due to their solubility in the extracellular fluid. Since the stratum corneum acts as a barrier for transdermal absorption, skin penetration of chemicals is expected to be slower than penetration through the gastrointestinal epithelial cells. However, the rapid transdermal absorption of low-molecular-weight water-soluble substances has been reported [31]. Another reason for reduced bioavailability following extravascular administration is the metabolism that occurs during absorption, called the first-pass effect. Following oral administration, the first-pass effect occurs in the gastrointestinal tract and the liver. These gastrointestinal and hepatic first effects decrease the bioavailability of drugs with low metabolic stability to enzymes such as CYPs and carboxylesterases. During percutaneous absorption, the chemical can be metabolized by esterase in rat skin.

The main elimination mechanism of systemically exposed hexamethylenetetramine is considered renal excretion rather than enzymatic metabolism. It is also possible that enzymatic degradation may be insignificant during absorption following oral and transdermal application of hexamethylenetetramine.

In summary, it is expected that hexamethylenetetramine penetrates biological membranes through the paracellular pathway due to its low-molecular-weight water-soluble physicochemical properties and exhibits high bioavailability regardless of the route of administration due to its relatively high metabolic stability.

## 5. Conclusions

The present study is the first report on a simple and sensitive LC–MS/MS assay for the determination of hexamethylenetetramine in a biological matrix and the toxicokinetics including the bioavailability of hexamethylenetetramine. The accuracy and precision of the analytical method were validated, and the method was successfully applied to investigate the toxicokinetics of hexamethylenetetramine in rats. Following oral and percutaneous administration, most of the administered hexamethylenetetramine was absorbed into the systemic circulation system. The findings of this study are expected to be useful for further toxicokinetic research and risk assessment of hexamethylenetetramine.

## Figures and Tables

**Table 1:** Slopes, intercepts, and coefficients of determination of calibration curves.

Batch	Slope	Intercept	r^2^
1	1.17246	0.00120	>0.9999
2	1.34961	0.00145	>0.9999
3	1.04113	0.00005	>0.9999
4	1.14158	0.00070	>0.9999
5	1.17224	0.00161	>0.9999
Mean	1.17541	0.00100	-
SD	0.11129	-	-
CV (%)	9.46806	-	-

**Table 2:** Intra- and inter-day accuracy and precision of LLOQ (5 ng/mL) and QC samples (15–800 ng/mL) for hexamethylenetetramine in rat plasma.

Concentration (ng/mL)	Intra-Day (*n* = 4)		Inter-Day (*n* = 4)	
	Accuracy (%)	CV (%)	Accuracy (%)	CV (%)
800	100.0	1.8	101.4	1.7
80	98.0	2.6	99.0	4.4
15	97.4	4.3	98.4	4.3
5	92.5	3.3	102.5	4.3

**Table 3:** Average non-compartmental toxicokinetic parameters of hexamethylenetetramine obtained after iv injection in rats.

Parameter	0.2 mg/kg (*n* = 4)	0.5 mg/kg (*n* = 6)
t_1/2_ (h)	1.36 ± 0.3	1.26 ± 0.22
C_0_ (ng/mL)	324.7 ± 66.92	797.97 ± 244.75
AUC_last_ (ng·h/mL)	323.9 ± 40.25	839.76 ± 75.03
AUC_infinity_ (ng·h/mL)	339.04 ± 38.91	856.46 ± 73.1
Vd_ss_ (L/kg)	0.93 ± 0.16	0.95 ± 0.19
CL (mL/min/kg)	9.94 ± 1.22	9.79 ± 0.78

**Table 4:** Average non-compartmental toxicokinetic parameters of hexamethylenetetramine obtained after oral and percutaneous administration in rats.

Parameter	Oral	Percutaneous	
	0.5 mg/kg (*n* = 3)	0.15% (*n* = 5)	0.6% (*n* = 5)
t_1/2_ (h)	1.12 ± 0.19	1.69 ± 0.56	2.43 ± 0.58
C_max_ (ng/mL)	364.23 ± 58.56	181.37 ± 42.95	843.47 ± 384.56
T_max_ (h)	0.47 ± 0.29	3.6 ± 1.67	2.9 ± 2.07
AUC_last_ (ng·h/mL)	766.14 ± 59.57	778.53 ± 256.34	2965.78 ± 1111.59
AUC_infinity_ (ng·h/mL)	793.02 ± 76.32	838.33 ± 262.05	3115.06 ± 1058.45
F (%)	89.83 ± 6.98	78.91 ± 24.57	77.19 ± 26.3

## Data Availability

Not applicable.
